# Using innovative customer relationship management technologies to explore the business opportunities of an ageing population and provide better service

**DOI:** 10.1186/2193-1801-4-S2-O6

**Published:** 2015-11-27

**Authors:** Paul Tak-wing Tsui, Fred Cheong-fai Li, Aaron Hok-chung Pang, Wai-fan Cheng

**Affiliations:** School for Higher and Professional Education, Vocational Training Council, Hong Kong; Department of Business Administration, Hong Kong Institute of Vocational Education (Chai Wan), Hong Kong

## Background

The ageing population is becoming a major concern in Hong Kong. According to population projections by the Census and Statistics Department [1], the number of people aged 65 and over will increase significantly from 1.02 million in 2013 to 2.56 million in 2041 and around one in three people in Hong Kong will be elderly in 2041 [2]. The Third Quarter Economic Report [3] noted that: (1) many elderly of the next and future generations will be fitter, better educated and better informed, and they will want to stay active in the community and (2) some of this demographic plan for and take care of their own needs. This 'silver hair market' provides new business opportunities for many industries, such as financial services, tourism, care services, fitness and grooming, and health food products.

Customer relationship management (CRM) is the strategic use of information technology and people to manage customers' relationships with companies (marketing, sales, services and support) across the whole customer life cycle [4].

Existing CRM technologies include databases, software (such as analysis and website development tools) and security features [5]. Researchers [6–8] have noted how recent innovative technology developments (such as big data, mobile business and devices, cloud computing, search engines, online retail platforms, social media and networks) have further modified the CRM model and enhanced its capability to build relationships and loyalty with target customers [9]. The purpose of this research is to study how innovative CRM technologies can be used to explore the business opportunities presented by the ageing population and provide better service to this population.

## Methods

An initial framework is developed by extensive literature review. The final framework is further supported and finalised by in-depth interviews with industry practitioners and academics. Content analysis is used to analyse the collected data.

## Results

The results provide evidence that innovative technology (such as big data, mobile business and devices, social media and networks, and online retail platforms) can reveal the business opportunities of the ageing population and provide better service to this segment. For example, the use of big data can provide a better understanding of the needs of the 'silver hair market' and the mobile business and online retail platforms can ease their purchasing processes.

## Conclusions

The research findings can be applied across a range of industries to improve CRM for the ageing population.
